# Activation and degranulation of CAR-T cells using engineered antigen-presenting cell surfaces

**DOI:** 10.1371/journal.pone.0238819

**Published:** 2020-09-25

**Authors:** Qassim Dirar, Teal Russell, Lumei Liu, Sarah Ahn, Gianpietro Dotti, Shyam Aravamudhan, Laura Conforti, Yeoheung Yun

**Affiliations:** 1 Nanoengineering Department, Joint School of Nanoscience and Nanoengineering, North Carolina Agricultural and Technical State University, Greensboro, North Carolina, United States of America; 2 FIT BEST Laboratory, Department of Chemical, Biological, and Bioengineering, North Carolina Agricultural and Technical State University, Greensboro, North Carolina, United States of America; 3 Department of Microbiology and Immunology, Lineberger Comprehensive Cancer Center, University of North Carolina Chapel Hill, Chapel Hill, North Carolina, United States of America; 4 Division of Nephrology, Department of Internal Medicine, University of Cincinnati, Cincinnati, Ohio, United States of America; Peter MacCallum Cancer Centre, AUSTRALIA

## Abstract

Adoptive cell transfer of Chimeric Antigen Receptor (CAR)-T cells showed promising results in patients with B cell malignancies. However, the detailed mechanism of CAR-T cell interaction with the target tumor cells is still not well understood. This work provides a systematic method for analyzing the activation and degranulation of second-generation CAR-T cells utilizing antigen-presenting cell surfaces. Antigen-presenting cell surfaces composed of circular micropatterns of CAR-specific anti-idiotype antibodies have been developed to mimic the interaction of CAR-T cells with target tumor cells using micro-contact printing. The levels of activation and degranulation of fixed non-transduced T cells (NT), CD19.CAR-T cells, and GD2.CAR-T cells on the antigen-presenting cell surfaces were quantified and compared by measuring the intensity of the CD3ζ chain phosphorylation and the Lysosome-Associated Membrane Protein 1 (LAMP-1), respectively. The size and morphology of the cells were also measured. The intracellular Ca^2+^ flux of NT and CAR-T cells upon engagement with the antigen-presenting cell surface was reported. Results suggest that NT and CD19.CAR-T cells have comparable activation levels, while NT have higher degranulation levels than CD19.CAR-T cells and GD2.CAR-T cells. The findings show that antigen-presenting cell surfaces allow a quantitative analysis of the molecules involved in synapse formation in different CAR-T cells in a systematic, reproducible manner.

## Introduction

Clinical trials with CAR-T cells redirected to target the pan-B cell antigen CD19 have shown promising results in treating children and adults with B-cell acute lymphoblastic leukemia [1, 2]. In contrast, clinical trials with CAR-T cells in patients with solid tumors such as neuroblastoma, pancreatic cancer, and glioblastoma demonstrated limited success [3–5]. These discrepancies in clinical outcomes with CAR-T cells between liquid and solid tumors call for a thorough understanding of how CAR-T cells interact with tumor cells [6]. In particular, understanding how CAR molecules expressed by T cells form immune synapses upon engaging the antigen expressed by tumor cells may represent a significant steppingstone to design better CAR-T cells.

In a recent report, Davenport et al. showed that the immune synapse formation of CAR-T cells differs from its counterpart in non-transduced T cells engaging the antigen via the classic T-cell-receptor (TCR) [7]. CAR-T-cells do not form the three concentric domains, known as “bulls’ eye,” upon interacting with target cells. CAR-T cells lacked the aggregation of lck in the central supramolecular activation cluster (cSMAC) and spent less time interacting with target cells [8, 9]. Xiong et al. investigated the quality of the immune synapse formation in CAR-T cells by quantifying F-actin, aggregation of tumor antigen, perforin polarization, and distribution of the phosphorylated CD3ζ chain [10]. The study showed that CAR-T cell effectiveness correlates positively with the quality of the immune synapse formed.

Engineering micropatterned surfaces by microcontact printing are one effective way to study the immune synapse formation in T cells. Micropatterned surfaces have been used in immunological studies, as they can mimic micro-scale interactions in a cost and labor effective manner. Microcontact printing allows for the isolation of molecules participating in the formation of the immune synapse to understand their specific role better [11]. Doh and Irvine developed immune synapse arrays carrying anti-CD3 monoclonal antibody (mAb) micropatterns and reproduced the formation of the “bull’s eye” [12]. Shen et al. used different orientations of anti-CD3 and anti-CD28 micropatterns to study the activation and cytokine secretion of CD4^+^ T cells [13]. Tabdanov et al. used micropatterning to investigate the relationship between the TCR and LFA-1 in regulating the cytoskeletal organization [14], while Motsch et al. used micropatterning to quantify the interaction between TCR and Zap70 [15]. Here, we develop a systematic method to study activation and degranulation in CAR-T cells using micropatterned surfaces. We quantify the levels of activation and degranulation, the size and morphology, and the intracellular Ca^2+^ flux of NT and CAR-T cells upon engagement with the antigen-presenting cell surface. Results show that our proposed method can be a reliable one to study CAR-T cells’ interaction with target antigens and can be easily extrapolated to study other types of CAR-T cells.

## Materials and methods

### CAR-T cell preparation

As proof of concept, we have used CAR-T cells expressing either a CD19-specific CAR (CD19.CAR-T cells) or a GD2-specific CAR (GD2.CAR-T cells) in which in both cases the CAR encodes the CD28 costimulatory endodomain. CAR-T cells were generated as previously described [16]. Briefly, human peripheral blood mononuclear cells (PBMC) were isolated from discharged buffy coats (Gulf Coast Regional Blood Center, Houston, TX, US) and activated by plate-bound CD3 (1 ng/ml; Miltenyi Biotec, MA, US) and CD28 (1 ng/ml; BD Biosciences, NJ, US) in media composed of 45% RPMI 1640, 45% Click’s Media (Irvine Scientific, CA, US), 10% fetal bovine serum (Hyclone, PA, US), 1% L-glutamine (Invitrogen, CA, US), IL-7 (10 ng/ml; Peprotech, NJ, US) and IL-15 (5 ng/ml; Peprotech, NJ, US). Stimulated PBMCs were transduced with a g-retrovirus encoding the 2^nd^ generation CD19 or GD2 CAR on retronectin-coated plates (Takara Bio Inc, CA, US) and expanded for 10 days prior to analysis. The transduction efficiencies of CAR-T cells were analyzed at day or 12–14 of culture.

### Fabrication of antigen-presenting cell surfaces

The protocol for using microcontact printing to pattern proteins was previously described [13, 17, 18]. First, round coverslips (1.2 cm) (Fisher Scientific, NH, US) are washed with detergent for 15 minutes at 110°C. Coverslips are then sonicated in acid solution (50 mL dH_2_O, 2 mL HNO_3_, 6 mL HCl) for 1 hour, rinsed thoroughly with deionized (DI) water, and stored in pure ethanol. PDMS stamps with circular pillars (5 μm diameter x 5 μm spacing x 5 μm tall) (Research Micro Stamps, SC, US) were incubated in 250–300 μL (50 μg/mL) of target antigen overnight. To mimic the microscale contact of CAR-T cells with the antigen, we used the anti-CD3 mAb for TCR activation and the anti-idiotype Abs 233-4A and 1A7 for CD19 CAR and GD2 CAR activation, respectively. Following incubation, stamps were sequentially rinsed in 0.5% Tween-20 in PBS, PBS, and DI water, and dried with nitrogen gas. The coverslips are removed from ethanol, dried with nitrogen gas, and treated with plasma for 3 minutes using Harrick Plasma Cleaner (Harrick Plasma, NY, US) with plasma power set at high. Then, stamps were immediately brought into contact with the plasma-treated coverslips for 5 minutes. Stamps were gently removed, and coverslips were washed with PBS to remove unbound protein. Lastly, coverslips were incubated with 40 μL of recombinant human ICAM-1Fc chimera protein (R&D Systems, MN, US) at a concentration of 2 μg/mL for 2 hours and rinsed with PBS. Control coverslips were prepared by incubating 40 μL of recombinant human ICAM-1Fc chimera protein (R&D Systems, MN, US) at a concentration of 2 μg/mL for 2 hours. Control coverslips and coverslips with micropatterns were placed in a 24-well plate for cells to be deposited on them. For live imaging studies, micropatterns were visualized by pre-labeling with Alexa Fluor 568 antibody labeling kit (Thermofisher Scientific, MA, US) following the manufacturer's protocol.

### T cell interaction with antigen-presenting cell surface

Cryopreserved cells were thawed and cultured in complete medium (1:1 ratio of RPMI-1640 (GE Healthcare Bio-Sciences, PA, US) and Click’s medium (IrvineScientific, CA, US) containing 10% FBS (Atlanta Biologicals, GA, US), 1% glutamine (Hyclone Laboratories, PA, US), 1% P/S (Hyclone Laboratories, PA, US), 10 ng/mL IL-7 (PeproTech, NJ, US), and 5 ng/mL IL-15 (PeproTech, NJ, US) at 37°C and 5% CO_2_ for 5 days. On day 5, cells were counted, normalized for transduction efficiency, and resuspended in a fresh, complete medium. Cells were seeded to the 24-well plate at a density of 1.5x10^5^ cells/well. Samples were incubated at 37°C and 5% CO_2_ for 30 minutes for NT and 10 minutes for CAR-T cells. Half of the media volume was carefully removed from each well, and 525 μL of 4% PFA in PBS (Thermofisher Scientific, MA, US) was gently added to the side of each well. Cells were incubated for 15 minutes, then 350 μL of liquid was removed, and 1.05 mL of permeabilization buffer (0.1% Triton X-100 in PBS) was added. Cells were incubated for another 10 minutes, then washed three times with 500 μL of PBS (5 min/wash on a rocker). PBS was removed, and 500 μL of blocking solution (4% BSA (Sigma Aldrich, MO, US) in PBS) was added to each well. Cells were incubated for 1–2 hours in blocking solution followed by washing and incubation with 350 μL of goat anti-mouse Alexa Fluor (AF) 568 secondary Ab (2 μg/mL) for 30 minutes to label the micropatterns. Cells were washed five times with PBS, then incubated with 350 μL of primary Ab (8 μg/mL p-CD3ζ-AF488, 8 μg/mL CD107a-AF405 in blocking solution) for 1 hour to label the phosphorylated -CD3ζ chain (p-CD3ζ) and the LAMP1/CD107a, respectively. Cells were washed three times with PBS and incubated with 500 μL of 4% PFA in PBS for 10 minutes. Finally, fixed samples were washed three times with PBS, mounted onto glass slides using Vectashield mounting medium (Vector Laboratories, CA, US), and visualized.

### Fluorescence imaging and analysis

Round coverslips mounted on glass slides were imaged using ZEISS LSM 710 two-photon confocal microscope (Carl Zeiss Microscopy, LLC, NY, US) and Zen 2.0 software. Images were acquired at 20x magnification (NA 0.8) air and (1024 x 1024 (425.1 μm × 425.1 μm)) resolution through consecutive scans of Brightfield, AF 405 (excitation 405 nm; 1 AU pinhole aperture), AF 488 (excitation 488 nm; 1 AU pinhole aperture), and AF 568 (excitation 561 nm; 1 AU pinhole aperture). For each sample, at least two field of views were randomly selected and imaged at the plane just above the micropattern. A minimum of 60 cells was imaged from each sample. Acquired images were analyzed using ImageJ following previously published protocols [19–22]. Cells were outlined manually, and the integrated density of each cell outline and its adjacent non-fluorescent area was measured across all channels using the “analyze/measure” command. Measurements of non-fluorescent regions were used as background measurements and were subtracted from the integrated density of the cell outline. Hence, the corrected total cell fluorescence (CTCF) was calculated using the following equation:

$$\mathrm{CTCF}={\mathrm{Integrated}\;\mathrm{density}}_{\mathrm{cell}\;\mathrm{outline}}–({\mathrm{Area}}_{\mathrm{cell}\;\mathrm{outline}}*{\mathrm{Mean}}_{\mathrm{background}})$$ (1)

By subtracting the area and the mean fluorescence of the surrounding regions, Eq (1) mitigates both the effect of cell size and background on the integrated density of the cell outline. The distribution of p-CD3ζ and LAMP1 was measured using the integrated density of cells because the integrated density can capture the dim and the bright regions in the cell outline compared to measuring the mean [19–22].

#### Measurement of cell morphology

Cell morphology was quantitatively measured using the circularity measurement in ImageJ. Circularity is a shape descriptor with values ranging from zero to one. A circularity value of 1 represents a perfect circle, while a value less than 1 represents more elongated shapes with 0 representing a perfect ellipse. The “Threshold” and “Analyze Particles” commands in ImageJ were used to quantify the morphology of cells. For thresholding, the “Default” method was applied. Cell’s circularity was measured according to the following equation:

$$\mathrm{Circularity}=4\pi \;\frac{{\mathrm{Area}}_{\mathrm{cell}\;\mathrm{outline}}}{{\left({\mathrm{Perimeter}}_{\mathrm{cell}\;\mathrm{outline}}\right)}^{2}}$$ (2)

### Live cell imaging

For live cell imaging, cells were labeled with Fluo-4, AM, cell-permeant kit (Thermofisher Scientific, MA, US). Cells were loaded with Fluo-4, AM, according to the manufacturer’s protocol. Briefly, a solution of 1mM of Fluo-4-AM was prepared by dissolving 50 μg of Fluo-4 AM in 45.6 μL DMSO. The solution was vortexed for 1 minute, sonicated for 1 minute, and centrifuged for 1 minute. The concentration of the solution was halved by adding 20% Pluronic F-127 DMSO. Again, the solution was vortexed, sonicated, and centrifuged. The cell suspension was loaded with Fluo-4 AM at a total concentration of 1 μM. Cells were left in the dark at room temperature for 20 min; then cells were centrifuged for 2 minutes at 200 rpm. The cell pellet was resuspended in an indicator-free Tyrode solution from Sigma Aldrich (MO, US). The solution was left for 20 minutes to allow for the complete de-esterification of AM esters. Within two hours, Ca^2+^ imaging was performed using the Olympus IX83 confocal microscope (Olympus Scientific Solutions Americas Inc., MA, US) and cellSens software. Images were acquired at 20x magnification (NA 0.45) air and (438.6 μm × 330.24 μm) resolution through consecutive scans of Brightfield, Fluo-4 AM (excitation 488 nm), and AF 568 (excitation 561 nm) at the fastest setting (~12 frames/minute) for 10 minutes. The Ca^2+^ flux was measured mean intensity of Fluo-4AM value. The control samples were NT, CD19.CAR-T cells and GD2.CAR-T cells on ICAM-1 surface.

### Statistical analyses

Statistical analysis was performed using GraphPad PRISM 8 (CA, USA). Data were represented as mean ± standard deviation (SD) and analyzed using Welch ANOVA with α = 0.05. Games-Howell post hoc tests were used to compare individual groups with statistical significance accepted at p < 0.05.

## Results and discussion

### Fabrication of antigen-presenting cell surfaces

**Fig 1:**
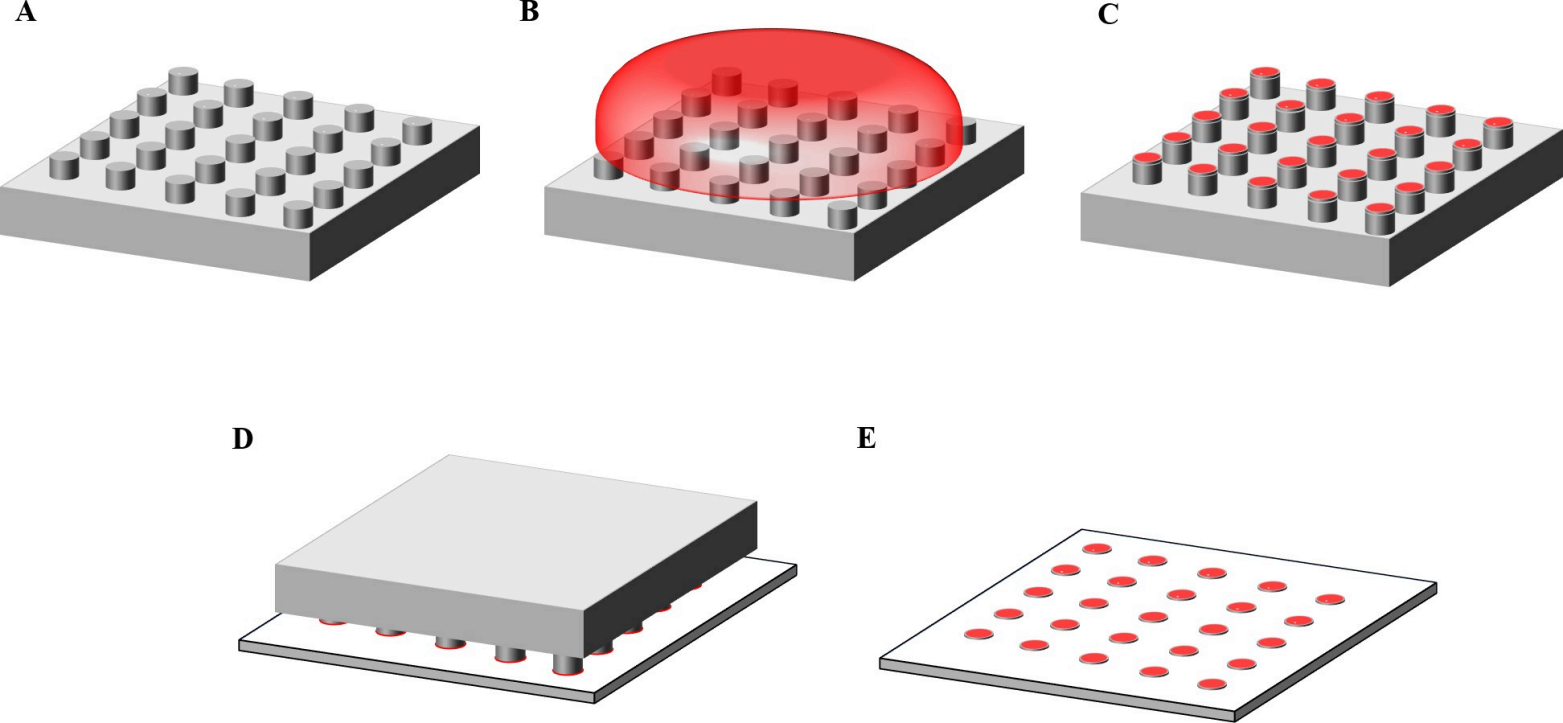
Schematic of steps of microcontact printing. (A) PDMS stamp with an array of posts as a pattern, (B) Incubation of the protein ink on the PDMS stamp, (C) Adsorption of the protein ink on the stamp, (D) Bringing the stamp onto contact with the glass coverslip, and (E) Transfer of the ink to the glass coverslip.

**Fig 2:**
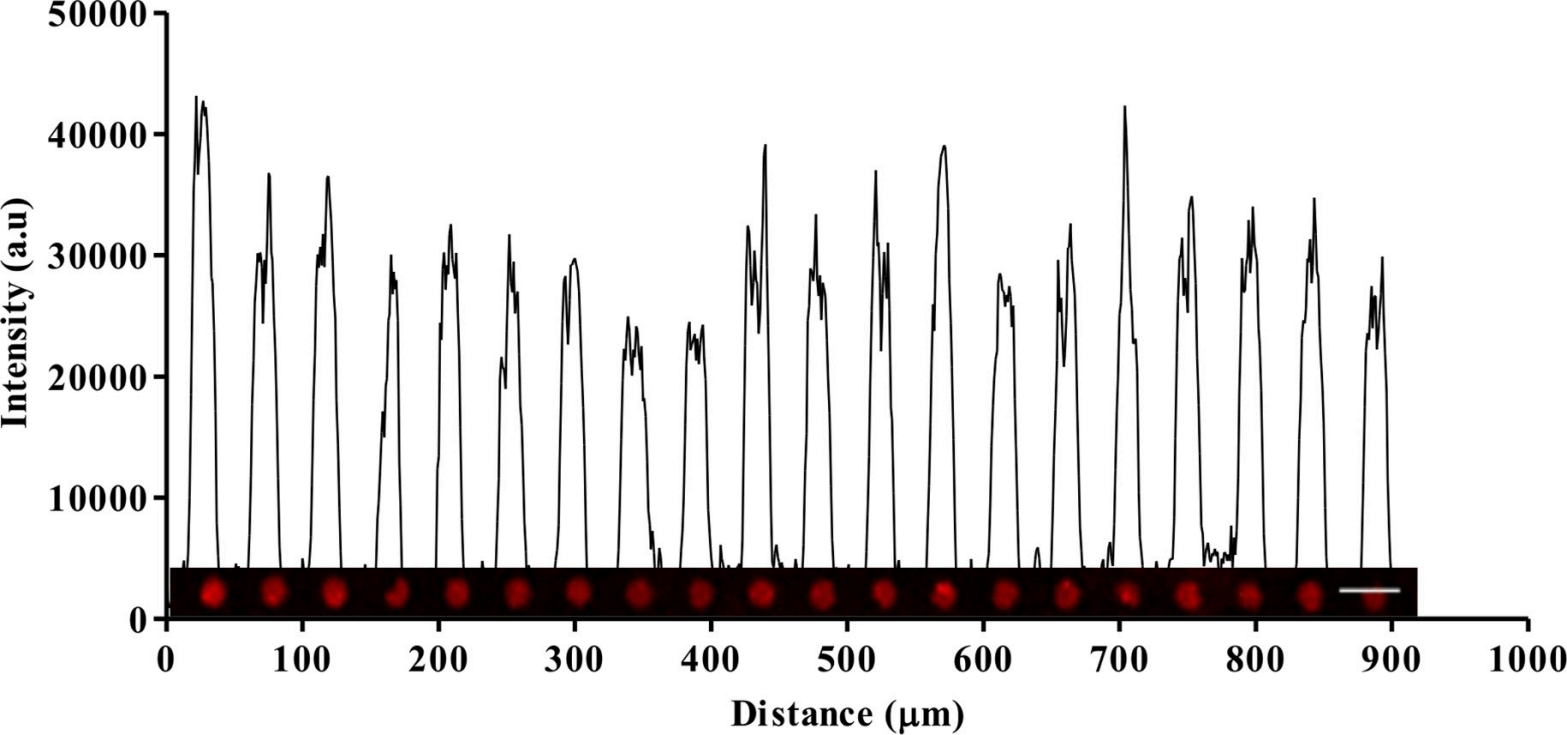
Intensity profile of anti-CD3 Ab micropatterns (50 μg/mL) labeled with 568 Rabbit Anti-Goat IgG (2 μg/mL) showing uniform intensity peaks indicating the uniform surface density of anti-CD3 Ab micropatterns. Scale bar 10 μm.

Antigen-presenting cell surfaces were fabricated using microcontact printing. Glass substrates were micropatterned with Abs using PDMS stamps, as shown in Fig 1. Printed micropatterns were fluorescently-labeled post pattern transfer rather than printing pre-labeled micropatterns to ensure that the Ab binding site is not blocked [12, 13]. In order to ensure consistent contact between the cells and the antigen-presenting cell surfaces, the surface density of the patterned micropatterns was inspected for uniformity [11, 13, 18]. The fluorescence intensity of the micropatterns correlated linearly with the concentration of the labeling Ab (S1 File), suggesting that a higher fluorescence intensity indicates a higher surface concentration of the micropatterns. Coverslips patterned with anti-CD3 Ab micropatterns was imaged using the ZEISS LSM 710 two-photon confocal microscope (Carl Zeiss Microscopy, LLC, NY, US) and analyzed with ImageJ (NIH, US). The surface density of the micropatterns was inspected by a plot profile of the fluorescence intensity of anti-CD3 Ab micropatterns (50 μg/mL) labeled with 568 Rabbit Anti-Goat IgG (2 μg/mL). The profile showed equal intensity peaks, indicating a uniform surface density of micropatterns, see Fig 2.

### Quantitative analysis of T cell activation

**Fig 3:**
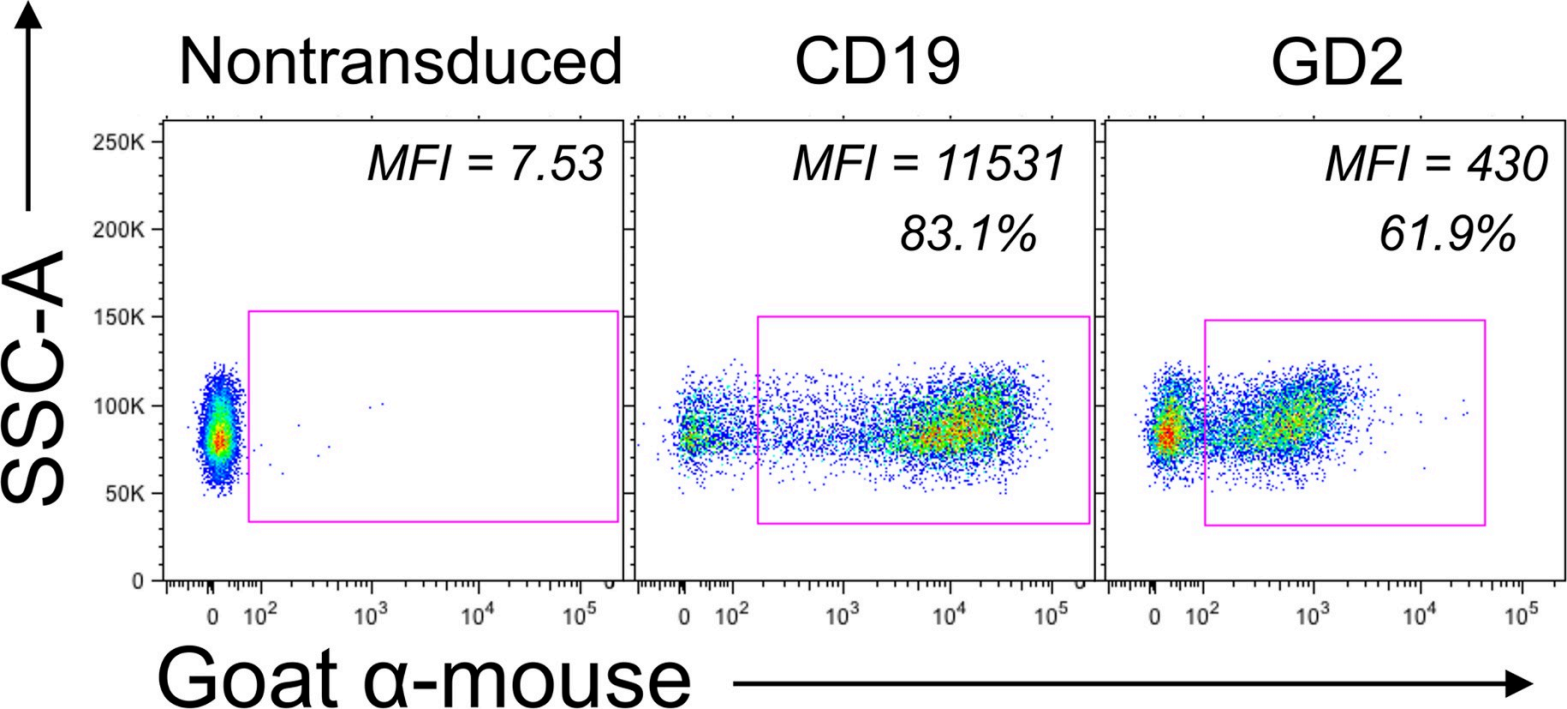
Transduction efficiencies for CD19.CAR-T cells and GD2.CAR-T cells represented by Median Fluorescence Intensity (MFI). Cells were stained with 233-4A (CD19 CAR target) and 1A7 (GD2 CAR target) anti-idiotype Abs followed by incubation with a goat-anti mouse secondary Ab. Control cells are represented by T cells (NT) following the same procedure of activation and expansion, but without the vector transduction.

**Fig 4:**
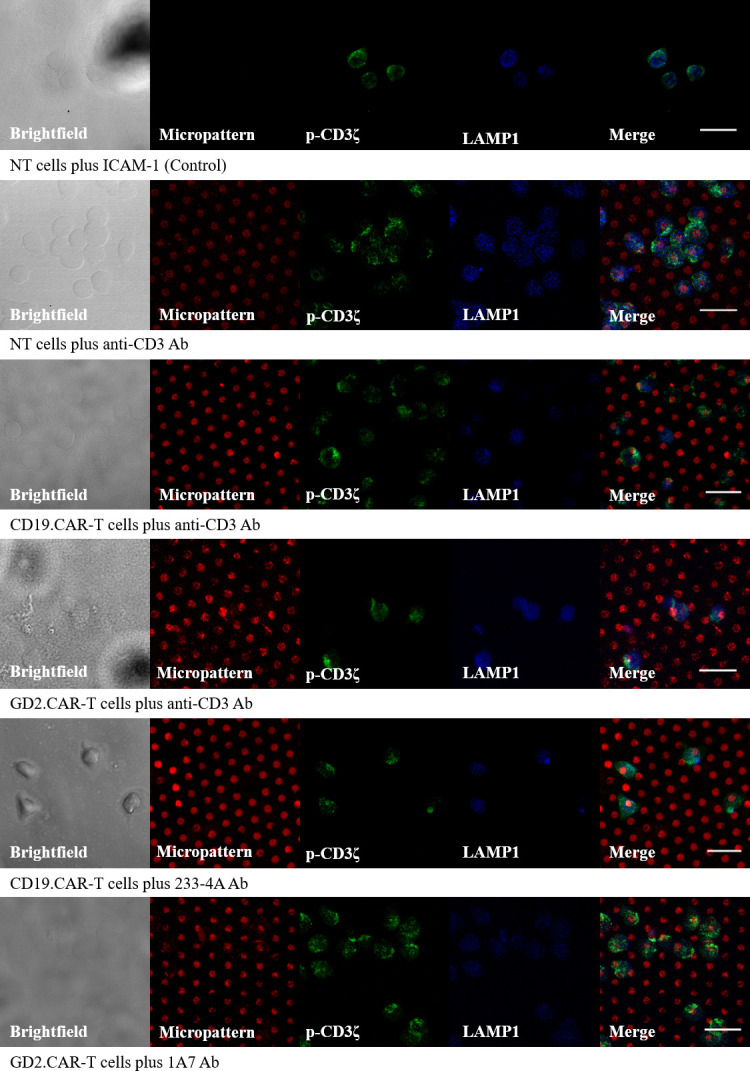
Activation and degranulation of NT and CAR-T cells on ICAM-1-coated surface (control) and antigen-presenting cell surfaces depicted by the aggregation of p-CD3ζ and CD107a. (A) Bright-field image, (B) micropatterns (5 μm) labeled with Alexa Fluor 568 Goat anti-mouse secondary Ab, (C) p-CD3ζ labeled with Alexa Fluor 488 primary Ab, (D) LAMP1 labeled with Alexa Fluor 405 primary Ab, and (E) image of BCD combined. Scale bar 20 μm.

The activation of NT, CD19.CAR-T cells, and GD2.CAR-T cells upon interacting with antigen-presenting cell surfaces was studied. Cells were incubated on substrates coated with ICAM-1 (control), anti-CD3 Ab (TCR target), and anti-idiotype Ab (CAR target). The transduction efficiencies of CAR-T cells were assessed at day 12–14 of culture, see Fig 3. CD19.CAR-T cells showed higher transduction efficiency than GD2.CAR-T cells. Higher transduction efficiency translates to more CAR molecules on the cell surface, meaning that CD19.CAR-T cells had more CAR molecules than GD2.CAR-T cells. The number of CD19.CAR-T cells was normalized to offset the discrepancy in transduction efficiencies. CD19.CAR-T cells were diluted with NT to normalize the number of CAR-T cells in each population. Normalization eliminated the possibility of variation in response solely because of the higher number of CAR molecules in CD19.CAR-T cells. Upon incubating cells on the antigen-presenting cell surfaces, cells recognized the micropatterns resulting in activation and degranulation. NT, CD19.CAR-T cells, and GD2.CAR-T cells were incubated on anti-CD3 Ab micropatterns to test activation through the native TCR. Furthermore, CD19.CAR-T cells and GD2.CAR-T cells were incubated with their respective target anti-idiotype Ab (233-4A and 1A7) to monitor the activation through the CAR. For control samples, cells were incubated on surfaces coated with ICAM-1 only. The aggregation of p-CD3ζ at the contact point with micropatterns indicated cell activation through the native TCR and the CAR [23], see Fig 4. The degree of aggregation varied depending on the cell type and the substrate. NT had a high degree of p-CD3ζ aggregation. CD19.CAR-T cells and GD2.CAR-T cells also showed some aggregation of p-CD3ζ when activating through the TCR or the CAR. The aggregation of p-CD3ζ suggests that the antigen-presenting cell surfaces successfully activated NT and CAR-T cells upon contact.

**Fig 5:**
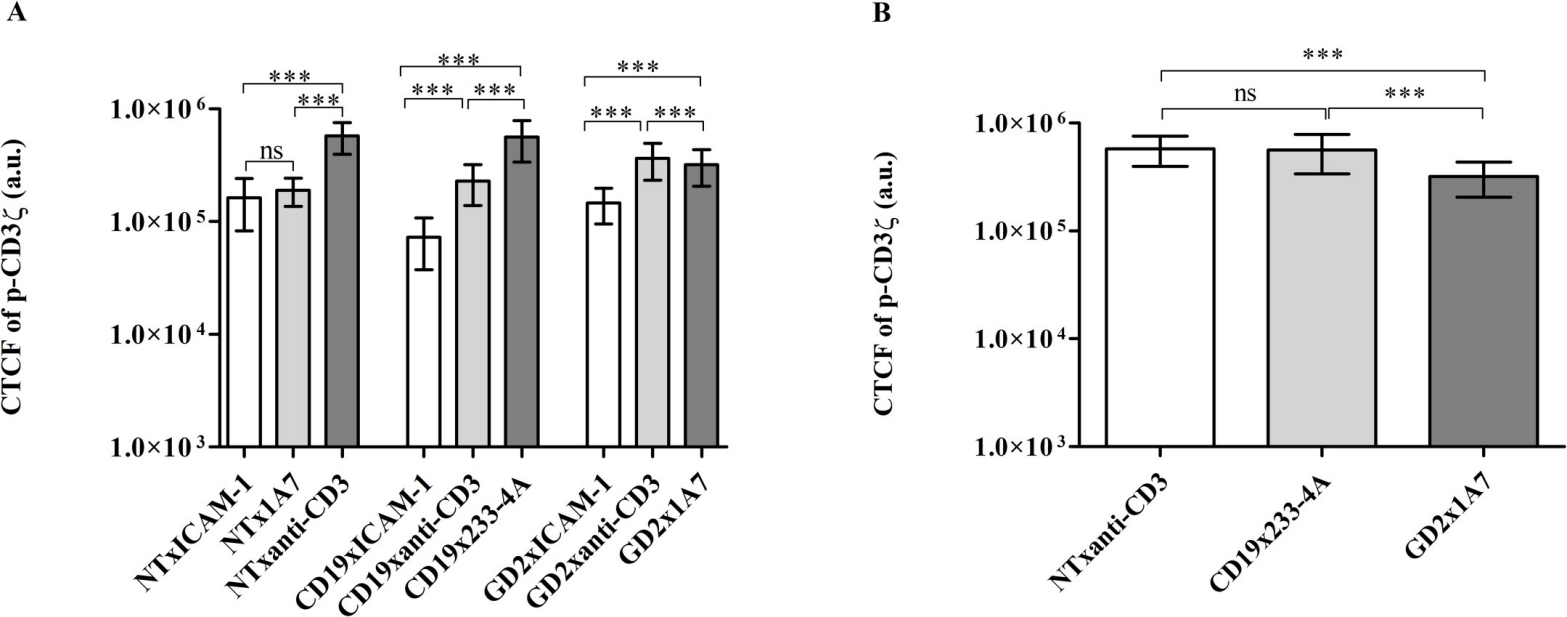
Comparison of p-CD3ζ levels of NT and CAR-T cells upon engagement with antigen-presenting cell surfaces. (A) Semilog plot of the corrected total cell fluorescence (CTCF) levels of Alexa Fluor 488-labeled p-CD3ζ in NT, CD19.CAR-T cells, and GD2.CAR-T cells on ICAM-1-coated surfaces (control) and micropatterns of anti-CD3 Ab and respective target anti-idiotype Abs. (B) Semilog plot of the comparison of CTCF levels of Alexa Fluor 488-labeled p-CD3ζ in NT, CD19.CAR-T cells, and GD2.CAR-T cells on micropatterns on respective target anti-idiotype Ab. Bars refer to the mean of CTCF, and the error bars refer to the standard deviation; ns not significant, *** p<0.001 (Welch ANOVA test, α = 0.05). Data for each sample were acquired from at least two fields of view (n >2) with all imaged cells included in the analysis (n >60). Data were pooled from three independents experiments.

The aggregation of p-CD3ζ was quantified by measuring the intensity of the AF488-labeled p-CD3ζ. Levels of activation in NT and CAR-T cells were compared to inspect the reported differences in activation between TCR and CAR engagement [8, 9, 12]. The corrected total cell fluorescence (CTCF) values of p-CD3ζ labeled with Alexa Fluor 488 for cells on control and target surfaces are provided in Fig 5. Activation levels of NT and CAR-T cells on ICAM-1-coated surfaces were similar since ICAM-1 is an adhesion molecule, and both NT and CAR-T cells interact similarly with adhesion molecules. NT did not show significant activation when treated with 1A7 Ab micropatterns suggesting that the response to the 1A7 Ab is CAR specific. As expected, NT and CAR-T cells showed higher levels of p-CD3ζ when exposed to the anti-CD3 Ab micropatterns because they remain susceptible to activation via their native TCR [5, 7, 10, 12, 13, 24]. However, both CD19.CAR-T cells and GD2.CAR-T cells showed lower levels of p-CD3ζ upon activation through the native TCR as compared to NT, suggesting that CAR molecules may partially sequester the endogenous CD3ζ chains making them less available for the native TCR. Both CD19.CAR-T cells and GD2.CAR-T cells showed high levels of CTCF as compared to control, upon interacting with their respective anti-idiotype Ab suggesting that micropatterns of 233-4A and 1A7 Ab successfully activated the CAR. However, while CD19.CAR-T cells stimulated through the CAR showed similar levels of activation as NT stimulated via TCR engagement, GD2.CAR-T cells showed inferior activation via CAR as compared to CD19.CAR-T cells. CD19.CAR-T cells and GD2.CAR-T cells were normalized for transduction efficiency before testing, but in general, the mean fluorescence intensity (MFI) of CD19 CAR is higher than GD2 CAR suggesting that more CAR molecules of the CD19 CAR are expressed even if the transduction efficiency is equal, and thus, activation upon CAR stimulation is superior. The high degree of CD19 CAR aggregation observed here is consistent with previous studies showing that CD19 CAR receptor aggregates when engaged to the target [25].

### Quantitative analysis of T cell degranulation

**Fig 6:**
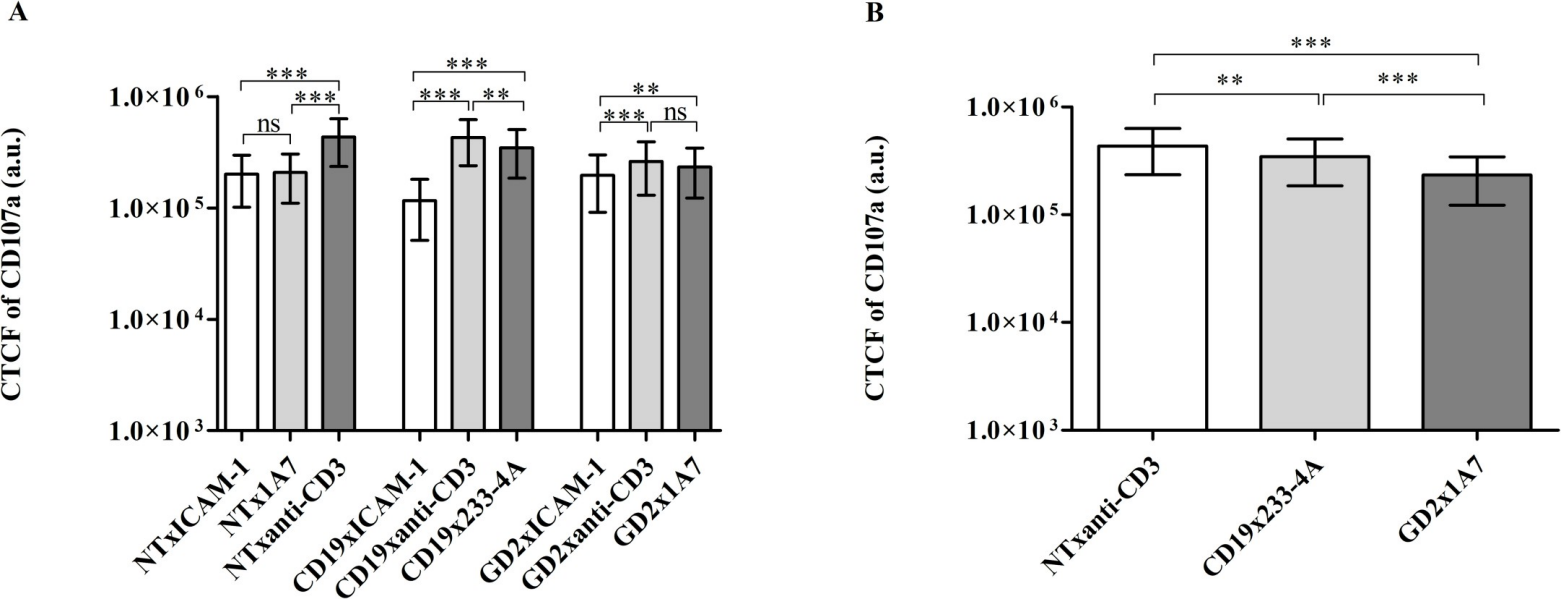
Comparison of CD107a levels of NT and CAR-T cells upon engagement with antigen-presenting cell surfaces. (A) Semilog plot of the corrected total cell fluorescence (CTCF) of Alexa Fluor 405-labeled LAMP1 in NT, CD19 and GD2 CAR-T cells on ICAM-1-coated surfaces (control) and micropatterns of anti-CD3 Ab and respective target anti-idiotype Abs, (B) Semilog plot of the comparison of CTCF of Alexa Fluor 405-labeled CD107a in NT, CD19, and GD2 CAR-T cells on surfaces micropatterned with respective target antigens, the bar refers to the mean of CTCF and the error bar refer to the standard deviation, ns not significant, ** p<0.02, *** p<0.001 (Welch ANOVA test, α = 0.05). Data were pooled from three experiments. Data for each sample was acquired from at least two field of views (n>2) with all imaged cells included in the analysis (n>60).

Degranulation of NT, CD19.CAR-T cells, and GD2.CAR-T cells upon interacting with antigen-presenting cell surfaces was quantified by measuring the intensity of the AF405-labeled LAMP-1/CD017a to examine the differences in degranulation between TCR and CAR engagement [8, 9, 12]. NT, CD19.CAR-T cells and GD2.CAR-T cells were incubated on anti-CD3 Ab micropatterns to analyze degranulation when cells are activated through the native TCR. Besides, CD19.CAR-T cells and GD2.CAR-T cells were incubated with their respective target anti-idiotype Ab (233-4A and 1A7) to inspect degranulation when cells are activated through the CAR. Fig 4 shows the expression of CD107a upon the interaction of cells with micropatterns. Cells showed different levels of CD107a expression depending on the cell type and the substrate. Fig 6 shows the corrected total cell fluorescence (CTCF) values of CD107a labeled with Alexa Fluor 405 for cells on control and target surfaces. NT and CAR-T cells interacting with ICAM-1-coated surfaces showed lower degranulation levels, as ICAM-1 is an adhesion molecule and does not induce degranulation independently [26]. NT did not show significant degranulation when activated with 1A7 Ab micropatterns suggesting that degranulation upon interacting with the 1A7 Ab is CAR specific. NT and CAR-T cells showed higher degranulation levels when incubated on anti-CD3 Ab micropatterns due to activation via the native TCR. Similar to the activation study, both CAR-T cells showed lower levels of degranulation compared to NT upon activating through the native TCR. Degranulation in NT activated with anti-CD3 Ab micropatterns increased by two-folds compared to control samples. CD19.CAR-T cells showed a three-fold increase in degranulation when activated by anti-CD3 Ab micropatterns, while GD2.CAR-T cells showed a one-fold increase. Both CD19.CAR-T cells and GD2.CAR-T cells upon interacting with their respective anti-idiotype Ab showed three-folds and one-fold increase in degranulation, respectively. The increase in degranulation suggests that micropatterns of 233-4A and 1A7 Ab successfully induced degranulation via the CAR. CD19.CAR-T cells showed higher degranulation levels when activated through the native TCR as compared to being activated through the CAR. Previous studies have already shown that CD19.CAR-T cells experience higher stimulation when stimulated through the TCR [27]. On the other hand, GD2.CAR-T cells showed similar levels of degranulation, whether stimulated through the TCR or the CAR.

The profiles of p-CD3ζ and CD107a levels (Figs 5B and 6B) of NT stimulated through the TCR and CD19/GD2.CAR-T cells stimulated through the CAR are similar. For both markers, the response of NT stimulated through the native TCR was higher than CAR-T cells. The higher level for both markers in NT could be attributed to the longer synapse duration in NT compared to the shorter synapse duration in CAR-T cells [7, 10, 24, 28].

### Morphology of cells interacting with antigen-presenting cell surfaces

**Fig 7:**
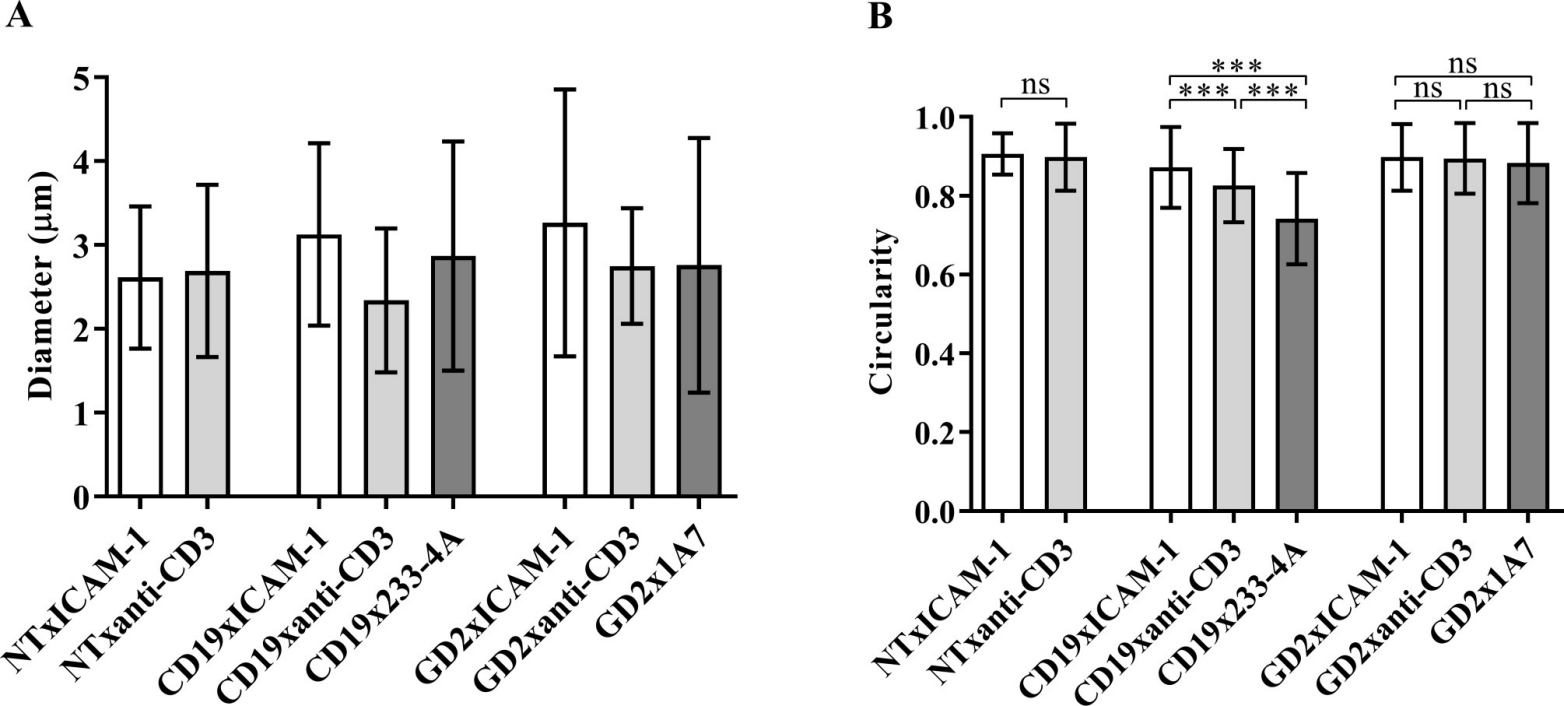
Comparison of size and morphology of NT, CD19.CAR-T cells, and GD2.CAR-T cells upon engagement with antigen-presenting cell surfaces. (A) Size of NT, CD19.CAR-T cells, and GD2.CAR-T cells on ICAM-1-coated surfaces (control) and micropatterns of anti-CD3 Ab and respective target anti-idiotype, (B) Cell morphology represented by the circularity of NT, CD19.CAR-T cells, and GD2.CAR-T cells on ICAM-1-coated surfaces (control) and micropatterns of anti-CD3 Ab and respective target anti-idiotype Abs.

The morphology and size of NT, CD19.CAR-T cells, and GD2.CAR-T cells were inspected to investigate whether different Ab micropatterns induced changes in cell morphology. In general, NT and CAR-T cells did not show a significant change in size after interacting with their respective target antigen-presenting cell surface. All cells had an average size of approximately 2.5 μm. Cell morphology was quantified using the circularity measurement in ImageJ. As shown in Fig 7, the morphology of NT and GD2.CAR-T cells did not change when interacting with either control or target surfaces. Both cell types exhibited an average circularity of 0.9. The circular morphology indicates that NT and GD2.CAR-T cells adhered to the surface after interacting with micropatterns [29]. The adhesion suggests that both NT and GD2.CAR-T cells recognized the substrate (anti-CD3 Ab and 1A7 anti-idiotype Ab) and interacted with it. On the contrary, CD19.CAR-T cells exhibited elongated morphology (circularity value less than 0.9), indicating that these cells displayed higher motility while interacting with micropatterns of anti-CD3 Ab and 233-4A anti-idiotype Ab [29–33]. The higher motility can be explained by the short engagement time of CAR-T cells with antigens, as shown in previous studies [7, 24].

### Live imaging

**Fig 8:**
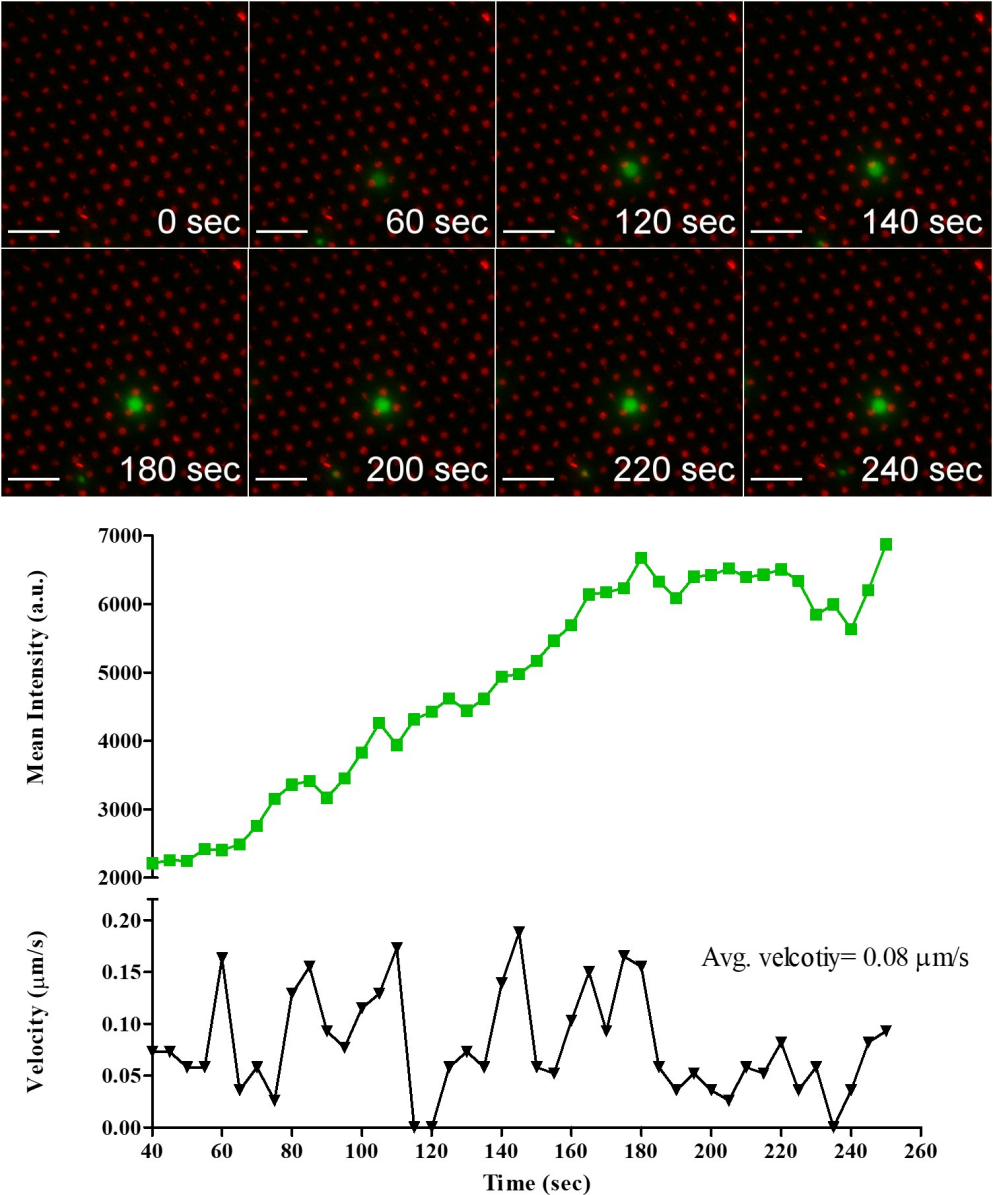
Temporal evolution of Ca^2+^ flux intensity in NT cell engaging anti-CD3 Ab micropatterns accompanied by profiles of Ca^2+^ flux intensity and cell velocity. Scale bar 20 μm.

To further confirm the activation of cells on the antigen-presenting cell surfaces, live imaging of single NT and CAR-T cell was conducted. We hypothesized that the antigen-presenting cell surfaces will activate both NT and CAR-T cells and will induce an increase in the intracellular Ca^2+^ flux. The intracellular Ca^2+^ flux in NT and CAR-T cells upon engagement with the antigen-presenting cell surface was reported by measuring the intensity of the green fluorescence of the cell-permeable Ca^2+^ sensitive dye Fluo-4-AM. The interaction of a single NT with anti-CD3 Ab micropatterns is shown in Fig 8; see S1 Movie. The NT came to a complete stop at 115 s, indicating that the cell is interacting with micropatterns [29]. The NT stopped for 5 seconds and then started moving again. The five seconds stop corresponded to an increase in the Ca^2+^ flux indicated by the increase in intensity. The NT stopped several times to interact with the pattern. Every stop correlated with an increase in Ca^2+^ flux.

**Fig 9:**
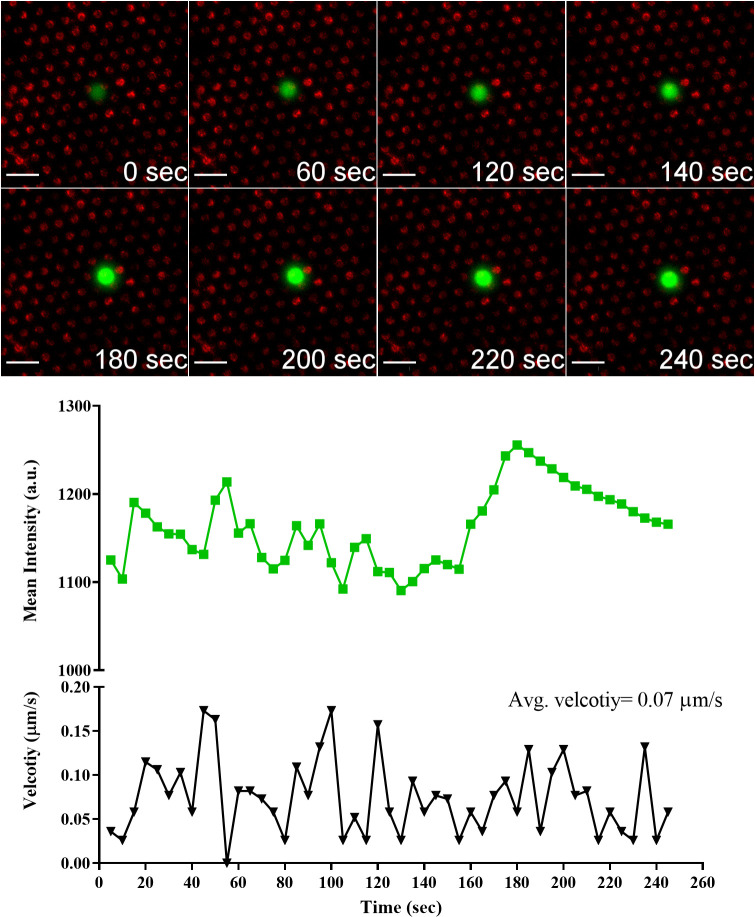
Temporal evolution of Ca^2+^ flux intensity in CD19.CAR-T cell engaging 233-4A Ab micropatterns accompanied by profiles of Ca^2+^ flux intensity and cell velocity. Scale bar 20 μm.

The interaction of a single CD19.CAR-T cell with 233-4A micropatterns is shown in Fig 9; see S2 Movie. The CD19.CAR-T cell came to a complete stop at 55 s. The complete stop corresponded to an increase in Ca^2+^ intensity, indicating that the CD19.CAR-T cell has recognized the micropattern. In the period between 130 and 160 s, the cell was precisely on top of the micropattern, and the intensity started increasing, reaching a maximum at 190 s.

**Fig 10:**
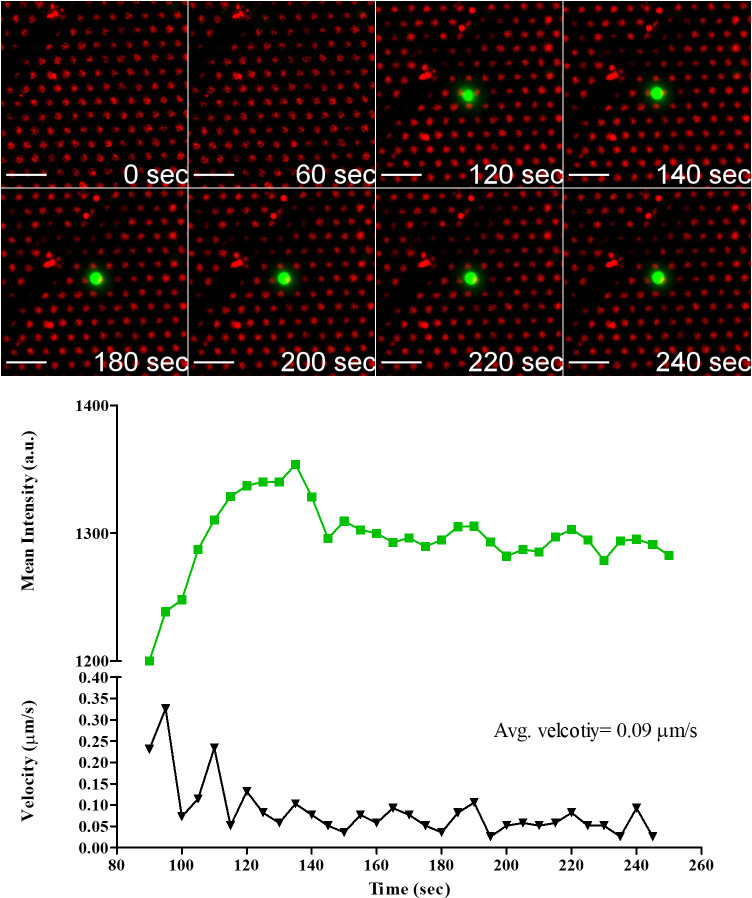
Temporal evolution of Ca^2+^ flux intensity in GD2.CAR-T cell engaging 1A7 Ab micropatterns accompanied by profiles of Ca^2+^ flux intensity and cell velocity. Scale bar 20 μm.

The GD2.CAR-T cell recognizing the 1A7 micropatterns demonstrated by the rapid increase in Ca^2+^ intensity, reaching a maximum at 130 s, see Fig 10. After that, the intensity decreased and stabilized. The constant intensity suggests that the cell was still engaged with the micropattern; see S3 Movie.

Looking at the Ca^2+^ flux in the three cells, the Ca^2+^ flux was higher for the NT cell than CD19 and GD2 CAR-T cells. The level of Ca^2+^ flux in both CAR-T cells was similar. The higher intensity level of the NT cell is in agreement with our previous results showing higher levels of p-CD3 ζ and LAMP1 in NT.

## Conclusion

In this study, we presented a systematic method to study activation and degranulation in CAR-T cells using antigen-presenting cell surfaces. The antigen-presenting cell surfaces successfully activated the cells and displayed uniform surface density. The levels of p-CD3ζ and CD107a in NT, CD19.CAR-T cells, and GD2.CAR-T cells were quantified. The level of activation of NT activating through the TCR and CD19.CAR-T cells activating through the CAR was comparable, while GD2.CAR-T cells showed inferior levels of activation through the CAR. NT activating via the TCR showed higher levels of degranulation than both CAR-T cells activating through the CAR. The results confirm that antigen-presenting cell surfaces successfully activated and induced degranulation in NT and CAR-T cells. Single-cell live imaging results were in agreement with our fixed-cells study showing that NT had a higher intracellular Ca^2+^ flux indicating higher activation than CAR-T cell. The insights from this study can help in designing better CAR-T cells and improve CAR-T cell therapy.

## Supporting information

S1 File Correlation of fluorescence and micropattern concentration. (DOCX)

S1 Movie NT cell interacting with anti-CD3 micropatterns. (AVI)

S2 Movie CD19.CAR-T cell interacting with 233-4A micropatterns. (AVI)

S3 Movie GD2.CAR-T cell interacting with 1A7 micropatterns. (AVI)

S1 Data (XLSX)

S2 Data (XLSX)

S3 Data (XLSX)
