# Supplementary material for: Activation and degranulation of CAR-T cells using engineered antigen-presenting cell surfaces
Source: PLoS One. 2020 Sep 25;15(9):e0238819. doi: 10.1371/journal.pone.0238819 (PMC7518621; doi:10.1371/journal.pone.0238819)

## Correlation of fluorescence and micropattern concentration

We tested printing unlabeled anti-CD3 at a concentration of 50 µg/mL and labeling it with AF-568 Goat anti-Mouse secondary IgG (2 µg/mL). This method ensures that the target antibody stays free to interact with the cell and avoids blocking the active binding site on the antibody. We established a calibration curve by measuring the intensity of a wide range of concentrations. We started by measuring the intensity of the stock solution and then diluted it by a factor of 2. We dipped a Q-tip in each solution and wiped it over a coverslip and imaged the samples with a fluorescence microscope keeping all the exposure parameters the same. **S1 Table** shows a summary of the concentrations used to establish the calibration curve. **S1 Fig** shows the fluorescent images of the different concentrations used.

**S1 Table.** **Concentrations used for establishing the calibration curve**.

| Sample | Concentration (mg/mL) |
| --- | --- |
| D0 | 2 |
| D1 | 1 |
| D2 | 0.5 |
| D3 | 0.25 |
| D4 | 0.125 |
| D5 | 0.0625 |
| D6 | 3.13E-02 |
| D7 | 1.56E-02 |
| D8 | 7.81E-03 |
| D9 | 3.91E-03 |
| D10 | 1.95E-03 |

**S1 Fig.** **Series of fluorescent images of different concentrations of AF-488 Rabbit anti-Goat IgG used to establish the calibration curve**. Scale bar 400 µm.


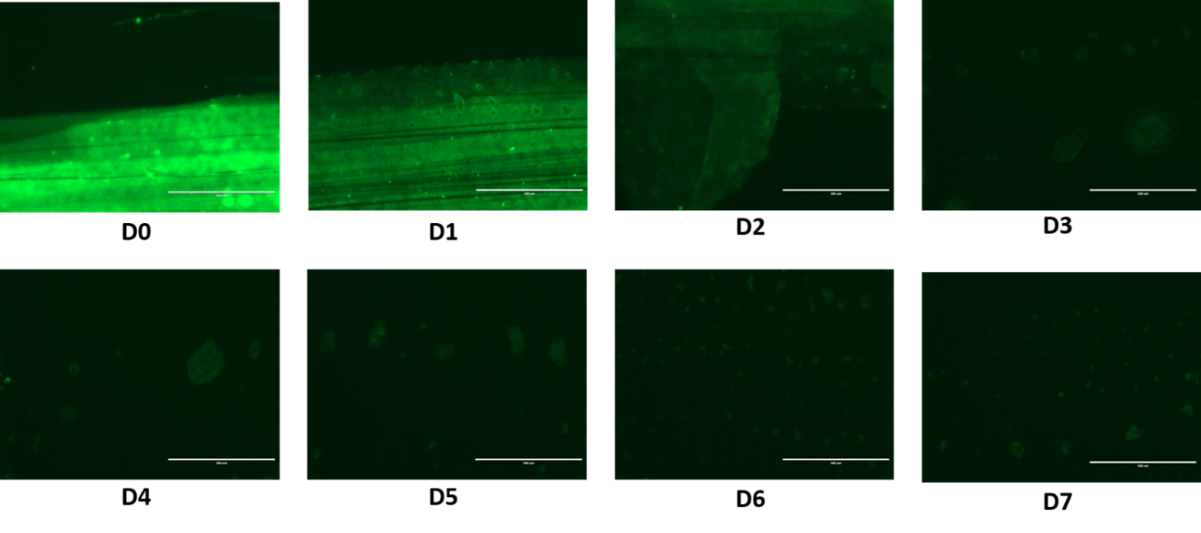


As expected, the fluorescence decreased with each concentration as the amount of fluorescent antibody is decreasing with further dilution. The signal of the last three dilutions was very low, so we did not include them in the analysis. We analyzed the image for each concentration using ImageJ. For each image, we measured the intensity of 5 different spots of the fluorescent layer along with the intensity of 3 different spots of the background and calculated the corrected total cell fluorescence (CTCF) for each spot according to equation (1).

$\text{CTCF = }{Integrated Density}_{\text{cell outline}}\text{ – (}\text{Area}_{\text{cell outline}}\text{*}\text{Mean}_{\text{background}})$ (1)

where the CTCF is the corrected total fluorescence, integrated density is the product of area and mean gray value measured by ImageJ, the area of the selected region is the area of the spot. This equation normalizes the fluorescence of spots with different areas to ensure that fluorescence measurement is not skewed by the area resulting in bigger spots having more fluorescence.

We calculated the average CTCF for each concentration sample. We plotted the average intensity for each concentration and fitted the data with a straight line.

**S2 Fig** shows an excellent correlation between the intensity and the concentration. Hence, the resulting equation can be used to quantify the concentration of antibody patterns produced by microcontact printing. Next, we measured the concentration of the patterned antibody dots by analyzing the image using the particle analysis feature in ImageJ. This feature allows the measurement of the size and areas of particles. Since our image has repetitive features of dots, they can be modeled as particles.

**S2 Fig.** **Intensity calibration curve for seven concentrations of AF-488 Rabbit anti-Goat IgG**. The linear regression equation shows an excellent linear correlation between concentration and fluorescence intensity.


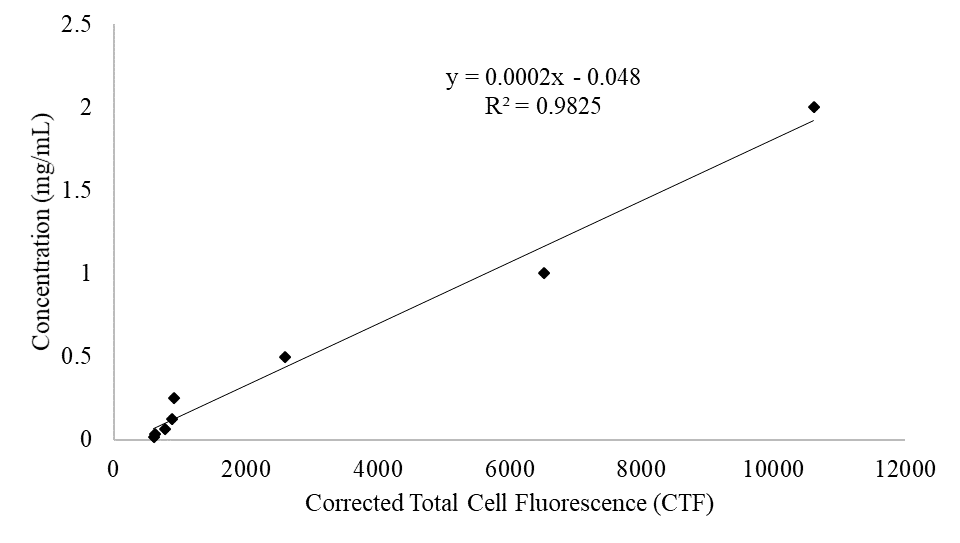


**S3 Fig** shows excellent replication of the patterned dots as particles. The average intensity of the pattern corresponded to a concentration of 137 µg/mL. The lower value might be due to the non-complete transfer of the protein from the PDMS stamp.

**S3 Fig.** **Patterns of AF-488 Rabbit anti-Goat IgG (green) (200** **µg/mL) modeled as particles by ImageJ**.


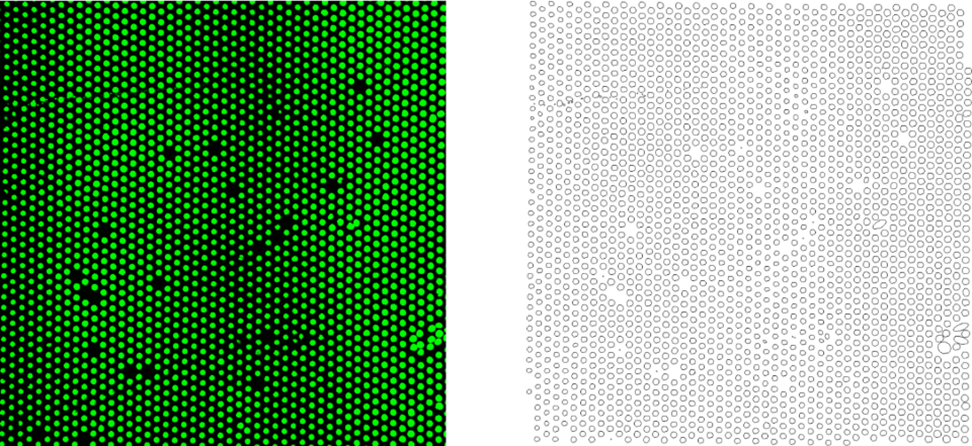

Supplement: S1 File — (DOCX) [file pone.0238819.s001.docx]
